# Supplementary material for: The contribution of common regulatory and protein-coding TYR variants to the genetic architecture of albinism
Source: Nat Commun. 2022 Jul 8;13:3939. doi: 10.1038/s41467-022-31392-3 (PMC9270319; doi:10.1038/s41467-022-31392-3)
Supplement: Supplementary file 1 — Supplementary Information [file 41467_2022_31392_MOESM1_ESM.pdf]

SUPPLEMENTARY INFORMATION

The contribution of common regulatory and protein-coding *TYR* variants to the genetic architecture of albinism

Vincent Michaud[1,2], Eulalie Lasseaux[1], David J. Green[3], Dave T. Gerrard[3], Claudio Plaisant[1], UK Biobank Eye and Vision Consortium, Tomas Fitzgerald[4], Ewan Birney[4], Benoît Arveiler[1,2], Graeme C. Black[3,5], Panagiotis I. Sergouniotis[3-6]

[1] Department of Medical Genetics, University Hospital of Bordeaux, Bordeaux, France.

[2] INSERM U1211, Rare Diseases, Genetics and Metabolism, University of Bordeaux, Bordeaux, France.

[3] Division of Evolution, Infection and Genomics, School of Biological Sciences, Faculty of Biology, Medicines and Health, University of Manchester, Manchester, UK.

[4] European Molecular Biology Laboratory, European Bioinformatics Institute (EMBL-EBI), Wellcome Genome Campus, Cambridge, UK.

[5] Manchester Centre for Genomic Medicine, Saint Mary's Hospital, Manchester University NHS Foundation Trust, Manchester, UK.

[6] Manchester Royal Eye Hospital, Manchester University NHS Foundation Trust, Manchester, UK.

SUPPLEMENTARY FIGURES

Supplementary Figure 1. Geographical distribution of selected *TYR* common variants and haplotypes.

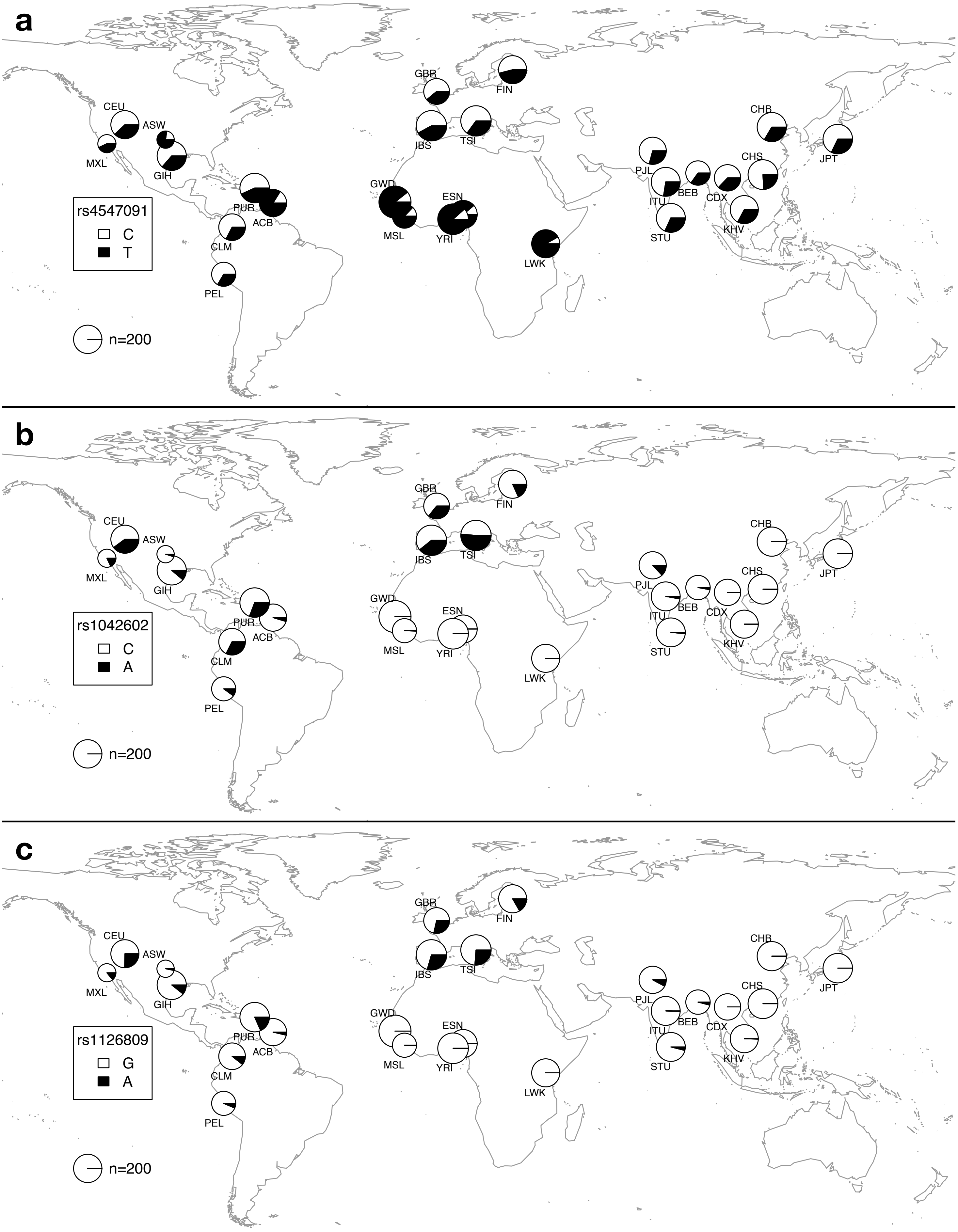

d

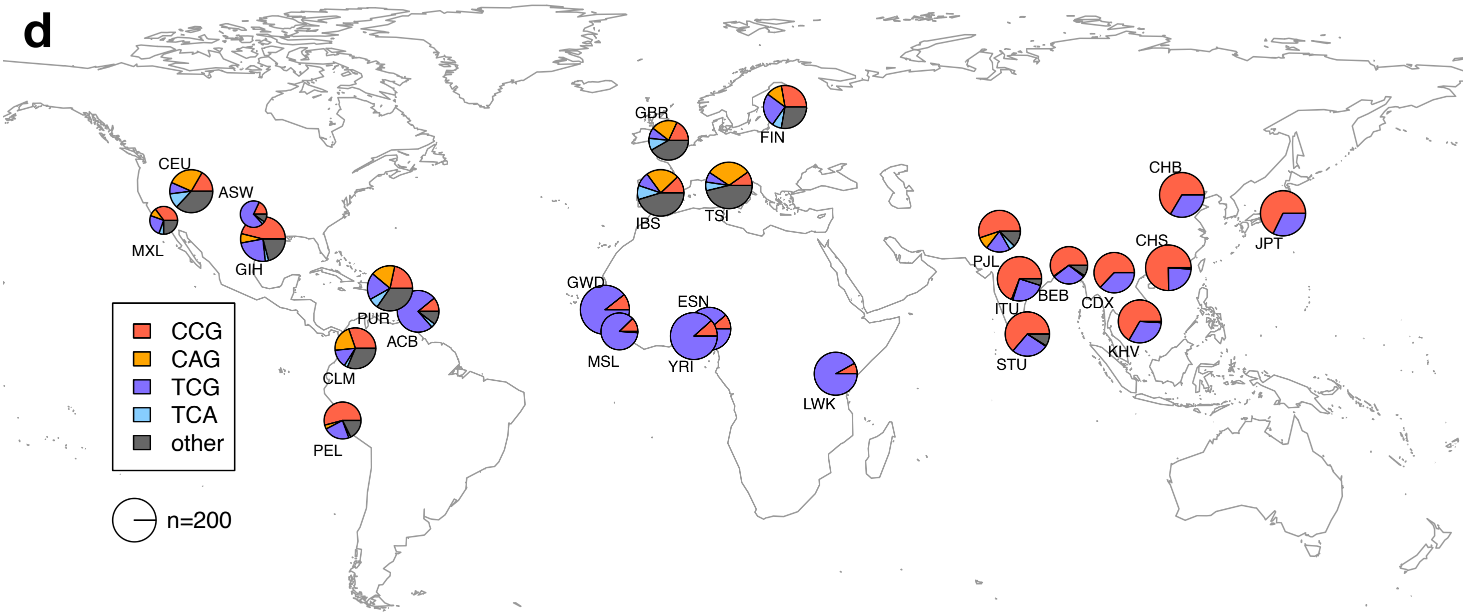

a-c. Geographical distribution of allele frequencies for the **(a)** *TYR* c.-301C>T [rs4547091], **(b)** *TYR* c.575C>A (p.Ser192Tyr) [rs1042602], and **(c)** *TYR* c.1205G>A (p.Arg402Gln) [rs1126809] variants across 1000 Genomes Project (phase 3) populations[1]. The R packages *maps*[2] and *plotrix*[3] were used to generate these maps; numerical data on sample size and allele frequencies are available at https://ldlink.nci.nih.gov/?tab=ldpop.[4] The allele frequencies of these variants across relevant subpopulations in the Simons Genome Diversity Project dataset[5] were comparable; these can be found in the Atlas of Variant Age resource (https://human.genome.dating/)[6]. This tool also provided insights into the estimated age of these changes:

- c.-301C>T [rs4547091]: T is predicted to be the ancestral allele; the estimated age of the derived C allele using a joint clock model was >1,000,000 years. This is a low-quality estimate that might be imprecise (quality score 0.2; values near 1 indicate high quality and values near 0 indicate low quality).
- c.575C>A [rs1042602]: C is the ancestral allele; the estimated age of the derived A allele using a joint clock model was ~23,000 years (quality score 0.9).
- c.1205G>A [rs1126809]: G is the ancestral allele; the estimated age of the derived A allele using a joint clock model was ~33,000 years (quality score 0.6).

Notably, the phylogeographic patterns for c.575C>A and c.1205G>A have been previously studied using a Bayesian coalescent approach; the estimated ages for c.575C>A and c.1205G>A were ~15,600 and ~29,400 years respectively.[7]

d. Geographical distribution of haplotype frequencies for the *TYR* c.[-301C;575C;1205G], c.[-301C;575A;1205G], c.[-301T;575C;1205G] and c.[-301T;575C;1205A] haplotypes (corresponding to [C;C;G], [C;A;G], [T;C;G] and [T;C;A] respectively) across 1000 Genomes Project (phase 3) populations[1]. The category 'other' contains two types of haplotypes: (i) 728 (out of a total of 5008; 15%) haplotypes from individuals who are heterozygous at two or more of the three variant positions (phase could not be inferred in these cases), and (ii) two further confirmed haplotypes, c.[-301C;575A;1205A] and c.[-301C;575C;1205A]), that were counted only twelve and two times, respectively. The R packages *maps*[2] and *plotrix*[3] were used to generate this map.

Population information and geographical coordinates were obtained from www.internationalgenome.org.

AFR (African): ACB (African Caribbean in Barbados); ASW (African Ancestry in Southwest US); ESN (Esan in Nigeria), GWD (Gambian in Western Division, The Gambia); LWK (Luhya in Webuye, Kenya); MSL (Mende in Sierra Leone); YRI (Yoruba in Ibadan, Nigeria).

AMR (American): CLM (Colombian in Medellin, Colombia); MXL (Mexican Ancestry in Los Angeles, California); PEL (Peruvian in Lima, Peru); PUR (Puerto Rican in Puerto Rico).

EAS (East Asian): CDX (Chinese Dai in Xishuangbanna, China); CHB (Han Chinese in Bejing, China); CHS (Southern Han Chinese); JPT (Japanese in Tokyo, Japan); KHV (Kinh in Ho Chi Minh City, Vietnam).

EUR (European): CEU (Utah Residents with Northern and Western European ancestry); FIN (Finnish in Finland); GBR (British in England and Scotland); IBS (Iberian populations in Spain); TSI (Toscani in Italy).

SAS (South Asian): BEB (Bengali in Bangladesh); GIH (Gujarati Indian from Houston, Texas); ITU (Indian Telugu in the United Kingdom); PJL (Punjabi in Lahore, Pakistan); STU (Sri Lankan Tamil in the United Kingdom).

Supplementary Figure 2. Impact of common *TYR* variants on central retinal thickness.

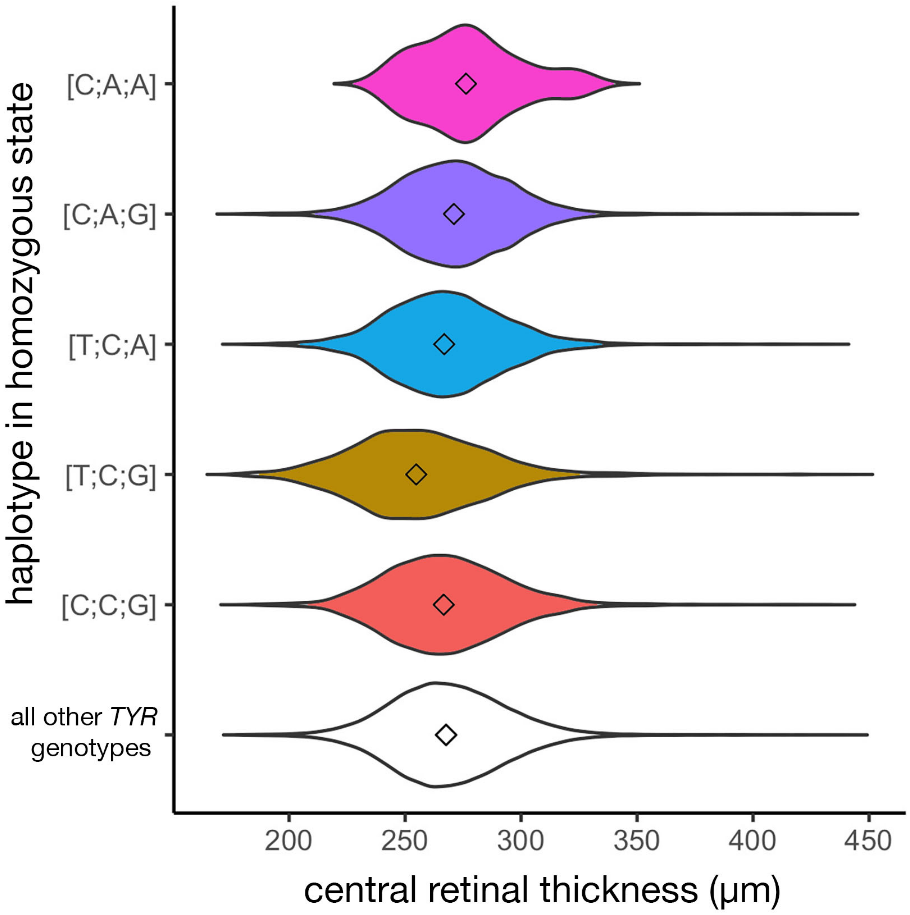

The distribution of central retinal thickness in UK Biobank participants who are homozygous for various *TYR* haplotypes is shown. The Kruskal-Wallis p-value was $< 2 \times 10^{-16}$ and further information including numerical data can be found in Supplementary Table 5.

[C;A;A] corresponds to homozygosity for *TYR* c.[-301C;575A;1205A], a genotype found to confer a high albinism risk;
[C;A;G] corresponds to homozygosity for *TYR* c.[-301C;575A;1205G];
[T;C;A] corresponds to homozygosity for *TYR* c.[-301T;575C;1205A], a genotype found to protect against albinism;
[T;C;G] corresponds to homozygosity for *TYR* c.[-301T;575C;1205G], a genotype found to protect against albinism;
[C;C;G] corresponds to homozygosity for *TYR* c.[-301C;575C;1205G].

It is noted that individuals with albinism tend to have increased central retinal thickness (due to underdevelopment of the fovea).

Supplementary Figure 3. Secondary analyses in selected subsets of the case and control cohorts confirm that common *TYR* variants form haplotypes that affect albinism risk.

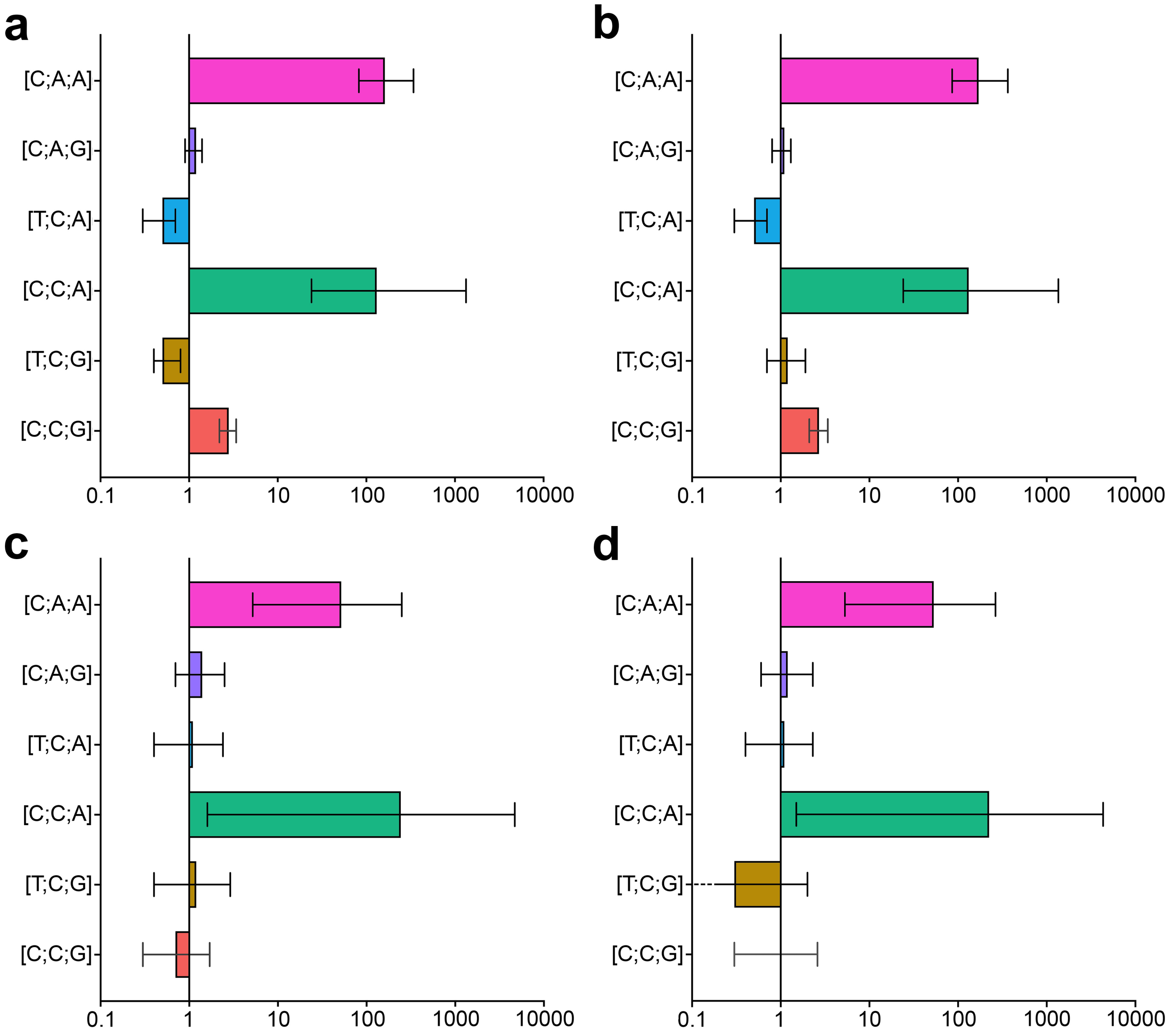

The x-axes are showing odds ratios corresponding to the probability of receiving a diagnosis of albinism for groups of individuals that carry selected *TYR* haplotypes in homozygous state. The length of each bar chart is proportionate to the value of the point estimate of each odds ratio; the relevant 95% confidence intervals are also shown. A $\log_{10}$ scale with 1 as the reference point is used; it is noted that an odds ratio >1 suggests an increased risk while an odds ratio <1 suggests a decreased risk.

Panel **a**, shows the results obtained following analysis of a mixed case cohort (including 1208 probands from the University Hospital of Bordeaux cohort and 105 cases from the 100K_GP dataset) and an inclusive control cohort (29,497 unrelated individuals from the 100K_GP dataset). This graph is identical to that shown in Figure 2b and is included here to facilitate comparison with the graphs in the other panels. Further information and relevant numerical data can be found in Supplementary Table 2.

Panel **b**, shows the results obtained following a similar analysis to that in panel **a**, but this time only focusing on individuals with predominantly European ancestries. Further information and relevant numerical data can be found in Supplementary Table 9.

Panel **c** shows the results obtained following analysis of cases and controls from the 100K_GP dataset only. Further information and relevant numerical data can be found in Supplementary Table 7.

Panel **d** shows the results obtained following a similar analysis to that in panel **c**, but this time only focusing on individuals with predominantly European ancestries. Further information and relevant numerical data can be found in Supplementary Table 8.

Overall, the findings of the sub-analyses discussed in panels **b, c** and **d** are well aligned with those of the primary analysis shown in panel **a** (and Figure 2b).

[C;A;A] corresponds to homozygosity for *TYR* c.[-301C;575A;1205A];
[C;A;G] corresponds to homozygosity for *TYR* c.[-301C;575A;1205G];
[T;C;A] corresponds to homozygosity for *TYR* c.[-301T;575C;1205A];
[C;C;A] corresponds to homozygosity for *TYR* c.[-301C;575C;1205A];
[T;C;G] corresponds to homozygosity for *TYR* c.[-301T;575C;1205G];
[C;C;G] corresponds to homozygosity for *TYR* c.[-301C;575C;1205G];
100K_GP corresponds to Genomics England 100,000 Genomes Project.

SUPPLEMENTARY TABLES

Supplementary Table 1. Cohort characteristics.

	**cases**	**controls**
number of probands	1,208 + 105 [a]	29,497
percentage of female probands	44% [b]	48%
percentage of probands with European ancestries [c]	84%	78%
genetic testing approach	gene panel testing or genome sequencing [a]	genome sequencing
probability of a proband carrying two or more rare HGMD-listed alleles [d] in the same albinism-related gene	38%	0.4%

[a] 1,208 probands with a diagnosis of albinism were identified through the University Hospital of Bordeaux Molecular Genetics Laboratory database; most of these cases underwent gene panel testing. A further 105 affected probands were identified in the Genomics England 100,000 Genomes Project dataset; these cases underwent genome sequencing using a similar pipeline to the control cohort.

[b] Additional analyses revealed that the observed enrichment in male probands in the case cohort is due to the contribution of X-linked forms of albinism.

[c] Ancestry was estimated using a questionnaire in most cases (1,208/1,313) and using principal component analysis in the remaining cases and in controls (see Methods).

[d] Refers to single nucleotide variants (or small insertions/deletions) that have minor allele frequency [MAF] < 1% and are labelled as disease-causing (DM) in the Human Gene Mutation Database (HGMD) v2021.2.

Supplementary Table 2. Risk of albinism in people carrying selected *TYR* haplotypes in homozygous state.

***TYR* haplotype (in homozygous state)**			**odds ratio [b]**	**95% confidence interval**	**p-value**
c.-301C>T [rs4547091] [a]	c.575C>A [rs1042602]	c.1205G>A [rs1126809]			
C (↓)	A	A	162	82-341	<0.00001
C (↓)	A	G	1.2	0.9-1.4	0.16
T (↑)	A	A	genotype not detected [c]		
T (↑)	A	G	genotype not detected [c]		
T (↑)	C	A	0.5	0.3-0.7	0.00003
C (↓)	C	A	131	24-1336	<0.00001
T (↑)	C	G	0.5	0.4-0.8	0.0007
C (↓)	C	G	2.8	2.2-3.4	<0.00001
**covariates**					
male gender			1.3	1.2-1.5	0.00002
presence of one rare risk genotype [d]			7.0	6.0-8.1	<0.00001
presence of two or more rare risk genotypes [d]			100	83-121	<0.00001
ancestry [e] (African)			4.1	2.9-5.5	<0.00001
ancestry [e] (American)			2.5	1.2-4.8	0.02
ancestry [e] (East Asian)			0.2	0.04-0.6	0.003
ancestry [e] (South Asian)			0.1	0.1-0.2	<0.00001

[a] The reference allele of this promoter variant (c.-301C) is associated with lower *TYR* expression and is shown as C (↓); the non-reference allele (c.-301T) is associated with higher gene expression and is shown as T (↑).

[b] Firth logistic regression was used to generate odds ratios and unadjusted p-values; 1,313 probands with albinism and 29,497 unrelated controls were included in this analysis.

[c] The [T;A;A] and [T;A;G] haplotypes were not detected in homozygous state in neither the case nor the control cohort; this is likely to be due to the strong linkage disequilibrium between the *TYR* c.-301T and c.575C alleles in European populations (D' 1.0, R^2 0.35 in British populations included in the 1000 Genomes Project[1]).

[d] One risk genotype corresponds to a heterozygous single nucleotide variant (or small insertion/deletion) with a minor allele frequency [MAF] < 1% and a "disease-causing" (DM) label in the Human Gene Mutation Database (HGMD) v2021.2; only variants in autosomal albinism-related genes were considered.

[e] Ancestry was estimated using a questionnaire in most cases (1,208/1,313) and using principal component analysis in the remaining cases and in controls (see Methods).

A plot of these results can be found in Figure 2b and in Supplementary Figure 3a.

Supplementary Table 3. Differences in the diagnostic yield of genetic testing and the prevalence of *TYR*-related albinism between individuals with presumed European and presumed African ancestries.

	**number of affected probands in the University Hospital of Bordeaux cohort**	**percentage of probands with a molecular diagnosis of albinism following clinical-grade genetic testing** [a]	**percentage of probands with a molecular diagnosis of *TYR*-related albinism following clinical-grade genetic testing** [b]	**percentage of individuals carrying the c.-301C>T [rs4547091] genotype** [c]
individuals with albinism and presumed European ancestries	1,032	70%	34%	30% (heterozygous; CT)<br>7% (homozygous; TT)
individuals with albinism and presumed African ancestries	156	89%	13%	17% (heterozygous; CT)<br>32% (homozygous; TT)

[a] Clinical-grade genetic testing refers to the genetic analysis that was performed at the University Hospital of Bordeaux Molecular Genetics Laboratory. This involved analyzing single-nucleotide changes, copy-number variants and the high-risk genotypes reported here (*i.e.* homozygosity for the c.[-301C;575A;1205A] and c.[-301C;575C;1205A] *TYR* haplotypes).

[b] The difference in the prevalence of *TYR*-related albinism between individuals with presumed European and individuals with presumed African ancestries was statistically significant (χ^2 p-value < 0.00001).

[c] The non-reference allele of the *TYR* promoter variant c.-301C>T is associated with higher gene expression. The increased prevalence of TT genotypes in individuals with presumed African ancestries may partly account for the relatively low prevalence of *TYR*-related albinism in these populations.

Supplementary Table 4. Visual acuity in UK Biobank participants carrying selected *TYR* haplotypes in homozygous state.

***TYR* haplotype (in homozygous state)**			**Number of people with visual acuity data in the UK Biobank**	**Median LogMAR visual acuity**	**Mean LogMAR visual acuity**
c.-301C>T [rs4547091] [a]	c.575C>A [rs1042602]	c.1205G>A [rs1126809]			
C (↓)	A	A	33	0.06	0.10
C (↓)	A	G	12,173	-0.01	0.03
T (↑)	A	A	0	not applicable	
T (↑)	A	G	0	not applicable	
T (↑)	C	A	7,413	-0.01	0.02
C (↓)	C	A	1	0.49	0.49
T (↑)	C	G	4,824	0.0	0.04
C (↓)	C	G	6,511	-0.01	0.03
all other *TYR* genotypes (including heterozygous)			79,558	-0.01	0.03

[a] The reference allele of this promoter variant (c.-301C) is associated with lower *TYR* expression and is shown as C (↓); the non-reference allele (c.-301T) is associated with higher gene expression and is shown as T (↑).

Given that:

- no UK Biobank participants were homozygous for the [T;A;A] and [T;A;G] haplotypes,
- only one UK Biobank participant had visual acuity assessment and was homozygous for the [C;C;A] haplotype,

these three genotype groups were excluded from further statistical analyses. The average ranks of the remaining six categories were compared using the Kruskal-Wallis method; a p-value of 8×10^{-11} was obtained. Pair-wise comparisons between homozygosity for [C;A;A] and the other categories revealed the following Benjamini-Hochberg adjusted p-values:
[C;A;A] vs [C;A;G], p-value 0.013;
[C;A;A] vs [T;C;A], p-value 0.010;
[C;A;A] vs [T;C;G], p-value 0.039;
[C;A;A] vs [C;C;G], p-value 0.013;
[C;A;A] vs all other *TYR* genotypes, p-value 0.011.

A plot of these results can be found in Figure 2c.

Supplementary Table 5. Central retinal thickness in UK Biobank participants carrying selected *TYR* haplotypes in homozygous state.

***TYR* haplotype (in homozygous state)**			**Number of people with optical coherence tomography (OCT) data in the UK Biobank**	**Median central retinal thickness**	**Mean central retinal thickness**
c.-301C>T [rs4547091] [a]	c.575C>A [rs1042602]	c.1205G>A [rs1126809]			
C (↓)	A	A	20	276	278
C (↓)	A	G	7,338	271	271
T (↑)	A	A	0	not applicable	
T (↑)	A	G	0	not applicable	
T (↑)	C	A	4,553	267	268
C (↓)	C	A	0	not applicable	
T (↑)	C	G	2,674	255	256
C (↓)	C	G	3,782	267	268
all other *TYR* genotypes (including heterozygous)			45,226	268	269

[a] The reference allele of this promoter variant (c.-301C) is associated with lower *TYR* expression and is shown as C (↓); the non-reference allele (c.-301T) is associated with higher gene expression and is shown as T (↑).

Given that no UK Biobank participants had OCT imaging of sufficient quality and were homozygous for the [C;C;A], [T;A;A] or [T;A;G] haplotypes, these three genotype groups were excluded from further statistical analyses. The average ranks of the remaining six categories were compared using the Kruskal-Wallis method; a p-value of $< 2 \times 10^{-16}$ was obtained. Pair-wise comparisons between homozygosity for [C;A;A] and the other categories revealed the following Benjamini-Hochberg adjusted p-values:
[C;A;A] vs [C;A;G], p-value 0.13;
[C;A;A] vs [T;C;A], p-value 0.10;
[C;A;A] vs [T;C;G], p-value 0.0007;
[C;A;A] vs [C;C;G], p-value 0.10;
[C;A;A] vs all other *TYR* genotypes, p-value 0.13.

A plot of these results can be found in Supplementary Figure 2.

Supplementary Table 6. Single-nucleotide variants used to calculate the genomic inflation factor lambda (λGC).

variant coordinates (GRCh38)	variant dbSNP rsID	variant consequence (MANE Select v0.95 transcript)	name of associated gene (HGNC)	χ^2 (Firth regression)	gnomAD v3.1.2 frequency in NFE [a]	CADD Phred score [b]	SpliceAI score
12-88507004-A-T	rs183924903	ENST00000644744.1: c.714+24T>A	*KITLG*	0.099	0.001705	0.002	0
3-52404411-C-T	rs150454901	ENST00000460680.6: c.1250+42G>A	*BAP1*	0.976	0.003101	0.017	0
3-119490496-A-G	rs142663532	ENST00000295588.9: c.798-55A>G	*POGLUT1*	0.314	0.00369	0.041	0
16-84016604-G-A	rs77876966	ENST00000299709.8: p.Leu359Leu	*SLC38A8*	0.864	0.05285	0.075	0.02
5-33944723-G-A	rs150473213	ENST00000296589.9: p.Val506Val	*SLC45A2*	9.828	0.0002205	0.077	0
15-58646011-A-G	rs201948093	ENST00000260408.8: c.735+44T>C	*ADAM10*	0.18	0.003235	0.191	0
6-397261-G-A	rs34318727	ENST00000380956.9: c.637+9G>A	*IRF4*	16.21	0.07294	0.193	0
3-133953627-T-C	rs199782188	ENST00000310926.11: c.724+36A>G	*SLCO2A1*	0.042	0.01306	0.222	0
1-154589737-T-G	rs115805812	ENST00000368474.9: c.2668+20A>C	*ADAR*	0.039	0.004735	0.247	0
10-98429811-C-A	rs11592273	ENST00000361490.9: p.Gly283Trp	*HPS1*	1.937	0.0758	0.337	0
15-58646039-G-A	rs138743329	ENST00000260408.8: c.735+16C>T	*ADAM10*	0.409	0.00178	0.385	0
3-69949177-C-G	rs181810413	ENST00000352241.9: c.880+9C>G	*MITF*	0.414	0.003381	0.553	0
15-55228733-T-C	rs368760836	ENST00000336787.6: c.240-21A>G	*RAB27A*	2.213	0.0001176	0.611	0
20-32230808-G-A	rs200896099	ENST00000375749.8: c.736-11G>A	*POFUT1*	0.143	0.001911	0.685	0.09
19-2114320-G-A	rs117387599	ENST00000643116.3: c.2424-18C>T	*AP3D1*	2.605	0.01143	0.698	0
15-27871136-T-C	rs41304383	ENST00000354638.8: c.2244+18A>G	*OCA2*	5.378	0.04011	0.831	0
4-54709427-C-T	rs72549293	ENST00000288135.6: p.Tyr373Tyr	*KIT*	0.983	0.0001029	0.894	0.03
12-52516840-G-A	rs144359915	ENST00000252242.9: p.Asn412Asn	*KRT5*	0.021	0.002307	0.94	0
9-21974641-C-G	rs45456595	ENST00000304494.9: c.150+37G>C	*CDKN2A*	0.177	0.003248	1.22	0.29
1-154597286-C-T	rs200651742	ENST00000368474.9: c.1935-19G>A	*ADAR*	0.069	0.00172	1.26	0
6-15524467-T-C	rs16876573	ENST00000344537.10: c.811+59A>G	*DTNBP1*	0.472	0.04261	1.9	0
4-174492145-C-T	rs17060532	ENST00000296522.11: c.663-51G>A	*HPGD*	0.016	0.04114	2.05	0

(continued)

variant coordinates (GRCh38)	variant dbSNP rsID	variant consequence (MANE Select v0.95 transcript)	name of associated gene (HGNC)	χ^2 (Firth regression)	gnomAD v3.1.2 frequency in NFE [a]	CADD Phred score [b]	SpliceAI score
6-131851119-A-G	rs200746177	ENST00000647893.1: c.431-23A>G	*ENPP1*	0.375	0.001323	2.26	0
6-15533192-C-A	rs75380691	ENST00000344537.10: c.667+48G>T	*DTNBP1*	1.93	0.005808	2.69	0.04
12-88506969-A-G	rs41283110	ENST00000644744.1: c.714+59T>C	*KITLG*	4.972	0.007056	2.76	0
2-219212539-T-C	rs114354921	ENST00000265316.9: c.1864-48A>G	*ABCB6*	0.039	0.005657	3.11	0
15-52372211-G-A	rs11637651	ENST00000399233.6: p.Ile910Ile	*MYO5A*	0.892	0.06795	3.24	0
12-52519946-G-A	rs11549951	ENST00000252242.9: p.Leu117Leu	*KRT5*	0.359	0.07463	3.29	0
12-52517611-G-A	rs149467228	ENST00000252242.9: p.Ala357Ala	*KRT5*	1.656	0.007071	3.56	0.01
19-2113468-G-A	rs146083008	ENST00000643116.3: c.2602-55C>T	*AP3D1*	0.029	0.02771	3.83	0.06
3-149155185-G-A	rs34197730	ENST00000296051.7: p.Thr493Thr	*HPS3*	0.601	0.03576	4.28	0.13
12-57751138-C-G	rs3211614	ENST00000257904.11: c.355-48G>C	*CDK4*	1.646	0.002073	4.51	0.06
12-88516544-G-T	rs182948635	ENST00000644744.1: c.364-54C>A	*KITLG*	0.02	0.006951	4.53	0
2-237540465-G-T	rs61737681	ENST00000264605.8: p.Ala408Ser	*MLPH*	<0.00001	0.03652	0.116	0
10-102065990-G-A	rs3737243	ENST00000299238.7: p.Gly172Gly	*HPS6*	6.705	0.1081	4.59	0

[a] gnomAD v3.1.2 frequency in NFE corresponds to minor allele frequency in the non-Finnish European subset of the Genome Aggregation Database (gnomAD) v3.1.2. We selected variants with an allele frequency broadly matching this of the genotypes studied in the case-control analysis.

[b] CADD corresponds to Combined Annotation-Dependent Depletion, an integrative annotation tool for genetic variants. CADD provides a ranking rather than a prediction and the PHRED-scaled score ranges from 1 to 99 (with higher values indicating more deleterious cases). For the λGC analysis we wanted to select variants that are unlikely to affect protein function and we only chose changes with a CADD PHRED-scaled score less than 5.

It is noted that λ_{median} was found to be 1.04. Given that this is close to the expected value of 1.00, it can be concluded that there is limited evidence of confounding by ancestry.

Supplementary Table 7. Risk of albinism in people carrying selected *TYR* haplotypes in homozygous state: sub-analysis of cases and controls from the Genomics England 100,000 Genomes Project cohort.

***TYR* haplotype (in homozygous state)**			**odds ratio** [b]	**95% confidence interval**	**p-value**
c.-301C>T [rs4547091] [a]	c.575C>A [rs1042602]	c.1205G>A [rs1126809]			
C (↓)	A	A	52	5.2-250	0.0036
C (↓)	A	G	1.4	0.7-2.5	0.3
T (↑)	C	A	1.1	0.4-2.4	0.8
C (↓)	C	A	243	1.7-4693	0.037
T (↑)	C	G	1.2	0.4-2.9	0.8
C (↓)	C	G	0.7	0.3-1.7	0.5
**covariates**					
male gender			0.9	0.6-1.3	0.6
presence of one rare risk genotype [c]			6.3	3.9-10	<0.00001
presence of two or more rare risk genotypes [c]			60	35-101	<0.00001
ancestry [d] (African)			1.5	0.5-4.4	0.5
ancestry [d] (American)			5.9	0.7-23	0.1
ancestry [d] (East Asian)			1.1	0.01-8.4	0.9
ancestry [d] (South Asian)			1.8	0.9-3.2	0.1

[a] The reference allele of this promoter variant (c.-301C) is associated with lower *TYR* expression and is shown as C (↓); the non-reference allele (c.-301T) is associated with higher gene expression and is shown as T (↑).

[b] Firth logistic regression was used to generate odds ratios and unadjusted p-values; 105 probands with albinism and 29,497 unrelated controls were included in this analysis.

[c] One risk genotype corresponds to a heterozygous single nucleotide variant (or small insertion/deletion) with a minor allele frequency [MAF] < 1% and a "disease-causing" (DM) label in the Human Gene Mutation Database (HGMD) v2021.2; only variants in autosomal albinism-related genes were considered.

[d] Ancestry was inferred using principal component analysis and five broad super-populations were projected (European, African, Admixed American, East Asian, South Asian) (see Methods).

A plot of these results can be found in Supplementary Figure 3b.

Supplementary Table 8. Risk of albinism in people carrying selected *TYR* haplotypes in homozygous state: sub-analysis of cases and controls from the Genomics England 100,000 Genomes Project cohort (European subset)[a].

***TYR* haplotype (in homozygous state)**			**odds ratio**[c]	**95% confidence interval**	**p-value**
c.-301C>T [rs4547091][b]	c.575C>A [rs1042602]	c.1205G>A [rs1126809]			
C (↓)	A	A	53	5.3-264	0.0035
C (↓)	A	G	1.2	0.6-2.3	0.56
T (↑)	A	A	genotype not detected[c]		
T (↑)	A	G	genotype not detected[c]		
T (↑)	C	A	1.1	0.4-2.3	0.9
C (↓)	C	A	224	1.5-4332	0.04
T (↑)	C	G	0.3	0.001-2	0.2
C (↓)	C	G	0.9	0.3-2.6	0.99
**covariates**					
male gender			0.9	0.6-1.4	0.6
presence of one rare risk genotype[d]			6	3.5-10	<0.00001
presence of two or more rare risk genotypes[d]			50	27-92	<0.00001

[a] Ancestry was inferred in cases and controls from the Genomics England 100,000 Genomes Project cohort using principal component analysis; only probands matching to the European subset of the 1000 genomes project (phase 3) dataset were included in this analysis (see Methods).

[b] The reference allele of this promoter variant (c.-301C) is associated with lower *TYR* expression and is shown as C (↓); the non-reference allele (c.-301T) is associated with higher gene expression and is shown as T (↑).

[c] Firth logistic regression was used to generate odds ratios and unadjusted p-values; 76 probands with albinism and 22,927 unrelated controls were included in this analysis

[d] One risk genotype corresponds to a heterozygous single nucleotide variant (or small insertion/deletion) with a minor allele frequency [MAF] < 1% and a “disease-causing” (DM) label in the Human Gene Mutation Database (HGMD) v2021.2; only variants in autosomal albinism-related genes were considered.

A plot of these results can be found in Supplementary Figure 3c.

Supplementary Table 9. Risk of albinism in people carrying selected *TYR* haplotypes in homozygous state: sub-analysis of cases and controls from the University Hospital of Bordeaux and the Genomics England 100,000 Genomes Project cohorts (European subsets) [a].

***TYR* haplotype (in homozygous state)**			**odds ratio** [c]	**95% confidence interval**	**p-value**
c.-301C>T [rs4547091] [b]	c.575C>A [rs1042602]	c.1205G>A [rs1126809]			
C (↓)	A	A	170	86-365	<0.00001
C (↓)	A	G	1.1	0.8-1.3	0.6
T (↑)	A	A	genotype not detected [c]		
T (↑)	A	G	genotype not detected [c]		
T (↑)	C	A	0.5	0.3-0.7	0.00003
C (↓)	C	A	132	24-1353	<0.00001
T (↑)	C	G	1.2	0.7-1.9	0.4
C (↓)	C	G	2.7	2.1-3.4	<0.00001
**covariates**					
male gender			1.4	1.2-1.6	<0.00001
presence of one rare risk genotype [d]			7.5	6.4-8.7	<0.00001
presence of two or more rare risk genotypes [d]			101	82-123	<0.00001

[a] Ancestry was estimated using a questionnaire in most cases (1,208/1,313) and using principal component analysis in the remaining cases and in controls (see Methods).

[b] The reference allele of this promoter variant (c.-301C) is associated with lower *TYR* expression and is shown as C (↓); the non-reference allele (c.-301T) is associated with higher gene expression and is shown as T (↑).

[c] Firth logistic regression was used to generate odds ratios and unadjusted p-values; 1,107 probands with albinism and 22,927 unrelated controls were included in this analysis.

[d] One risk genotype corresponds to a heterozygous single nucleotide variant (or small insertion/deletion) with a minor allele frequency [MAF] < 1% and a “disease-causing” (DM) label in the Human Gene Mutation Database (HGMD) v2021.2; only variants in autosomal albinism-related genes were considered.

A plot of these results can be found in Supplementary Figure 3d.

SUPPLEMENTARY REFERENCES

1. Auton, A. *et al.* A global reference for human genetic variation. *Nature* **526**, 68–74 (2015).

2. Brownrigg, R., Minka, T. P., Deckmyn, A., Becker, R. A. & Wilks, A. R. maps: Draw geographical maps R package version 3.4.0. (2021).

3. Lemon, J. *et al.* plotrix: Plotting functions R package version 3.8-2. (2021).

4. Alexander, T. A. & Machiela, M. J. LDpop: an interactive online tool to calculate and visualize geographic LD patterns. *BMC Bioinformatics* **21**, 1–4 (2020).

5. Mallick, S. *et al.* The Simons Genome Diversity Project: 300 genomes from 142 diverse populations. *Nature* **538**, 201–206 (2016).

6. Albers, P. K. & McVean, G. Dating genomic variants and shared ancestry in population-scale sequencing data. *PLOS Biol.* **18**, e3000586 (2020).

7. Hudjashov, G., Villems, R. & Kivisild, T. Global patterns of diversity and selection in human tyrosinase gene. *PLoS One* **8**, e74307 (2013).

ACKNOWLEDGEMENTS & CONSORTIA

We are very grateful to individuals and families who are included in the studied cohorts for their participation. We are also grateful to the clinical, research and support staff who helped with recruitment and to the teams who are managing these cohorts and the associated resources.

We acknowledge the contribution of the Genomics England Research Consortium. The members of this consortium are: John C. Ambrose, Prabhu Arumugam, Roel Bevers, Marta Bleda, Freya Boardman-Pretty, Christopher R. Boustred, Helen Brittain, Mark J. Caulfield, Georgia C. Chan, Greg Elgar, Tom Fowler, Adam Giess, Angela Hamblin, Shirley Henderson, Tim J. P. Hubbard, Rob Jackson, Louise J. Jones, Dalia Kasperaviciute, Melis Kayikci, Athanasios Kousathanas, Lea Lahnstein, Sarah E. A. Leigh, Ivonne U. S. Leong, Javier F. Lopez, Fiona Maleady-Crowe, Meriel McEntagart, Federico Minneci, Loukas Moutsianas, Michael Mueller, Nirupa Murugaesu, Anna C. Need, Peter O'Donovan, Chris A. Odhams, Christine Patch, Mariana Buongermino Pereira, Daniel Perez-Gil, John Pullinger, Tahrima Rahim, Augusto Rendon, Tim Rogers, Kevin Savage, Kushmita Sawant, Richard H. Scott, Afshan Siddiq, Alexander Sieghart, Samuel C. Smith, Alona Sosinsky, Alexander Stuckey, Mélanie Tanguy, Ana Lisa Taylor Tavares, Ellen R. A. Thomas, Simon R. Thompson, Arianna Tucci, Matthew J. Welland, Eleanor Williams, Katarzyna Witkowska, Suzanne M. Wood

We also acknowledge the contribution of the UK Biobank Eye and Vision Consortium. Members of this consortium are: Naomi Allen, Tariq Aslam, Denize Atan, Sarah Barman, Jenny Barrett, Paul Bishop, Graeme Black, Tasanee Braithwaite, Roxana Carare, Usha Chakravarthy, Michelle Chan, Sharon Chua, Alexander Day, Parul Desai, Bal Dhillon, Andrew Dick, Alexander Doney, Cathy Egan, Sarah Ennis, Paul Foster, Marcus Fruttiger, John Gallacher, David Garway-Heath, Jane Gibson, Jeremy Guggenheim, Chris Hammond, Alison Hardcastle, Simon Harding, Ruth Hogg, Pirro Hysi, Pearse Keane, Peng Tee Khaw, Anthony Khawaja, Gerassimos Lascaratos, Thomas Littlejohns, Andrew Lotery, Phil Luthert, Tom Macgillivray, Sarah Mackie, Bernadette Mcguinness, Gareth Mckay, Martin Mckibbin, Tony Moore, James Morgan, Richard Oram, Eoin O'Sullivan, Chris Owen, Praveen Patel, Euan Paterson, Tunde Peto, Axel Petzold, Nikolas Pontikos, Jugnoo Rahi, Alicja Rudnicka, Jay Self, Panagiotis Sergouniotis, Sobha Sivaprasad, David Steel, Irene Stratton, Nicholas Strouthidis, Cathie Sudlow, Zihan Sun, Robyn Tapp, Dhanes Thomas, Emanuele Trucco, Adnan Tufail, Ananth Viswanathan, Veronique Vitart, Mike Weedon, Cathy Williams, Katie Williams, Jayne Woodside, Max Yates, Jennifer Yip, Yalin Zheng.

This research was conducted using the UK Biobank Resource under projects 53144, 49978 and 2112.
