# Supplementary material for: The contribution of common regulatory and protein-coding TYR variants to the genetic architecture of albinism
Source: Nat Commun. 2022 Jul 8;13:3939. doi: 10.1038/s41467-022-31392-3 (PMC9270319; doi:10.1038/s41467-022-31392-3)
Supplement: Supplementary file 3 — Description of Additional Supplementary Files [file 41467_2022_31392_MOESM3_ESM.pdf]

Description of Additional Supplementary Files

File Name: Supplementary Data 1
Description: Findings in 1,208 probands with albinism who underwent clinical-grade genetic testing at the University Hospital of Bordeaux Molecular Genetics Laboratory.
